# Treatment with CB_**2**_ Agonist JWH-133 Reduces Histological Features Associated with Erectile Dysfunction in Hypercholesterolemic Mice

**DOI:** 10.1155/2013/263846

**Published:** 2013-11-03

**Authors:** Rodrigo Araujo Fraga-Silva, Fabiana Pereira Costa-Fraga, Fabrizio Montecucco, Younouss Faye, Silvia Quintao Savergnini, Sébastien Lenglet, François Mach, Sabine Steffens, Nikolaos Stergiopulos, Robson Augusto Souza dos Santos, Rafaela Fernandes da Silva

**Affiliations:** ^1^Institute of Bioengineering, Ecole Polytechnique Fédérale de Lausanne, Station 17, BM 5115, CH-1015 Lausanne, Switzerland; ^2^Division of Cardiology, Geneva University Hospitals, Faculty of Medicine, Foundation for Medical Researches, 64, Avenue de la Roseraie, 1211 Geneva, Switzerland; ^3^First Clinic of Internal Medicine, Department of Internal Medicine, University of Genoa, IRCCS Azienda Ospedaliera Universitaria San Martino - IST Istituto Nazionale per la Ricerca sul Cancro, 6 viale Benedetto XV, 16132 Genoa, Italy; ^4^Department of Physiology and Biophysics, Biological Science Institute, Federal University of Minas Gerais, Avenida Antonio Carlos, 6627, 31270-901 Belo Horizonte, MG, Brazil

## Abstract

Hypercholesterolemia is one of the most important risk factors for erectile dysfunction, mostly due to the impairment of oxidative stress and endothelial function in the penis. The cannabinoid system might regulate peripheral mechanisms of sexual function; however, its role is still poorly understood. We investigated the effects of CB_2_ activation on oxidative stress and fibrosis within the corpus cavernosum of hypercholesterolemic mice. Apolipoprotein-E-knockout mice were fed with a western-type diet for 11 weeks and treated with JWH-133 (selective CB_2_ agonist) or vehicle during the last 3 weeks. CB_2_ receptor expression, total collagen content, and reactive oxygen species (ROS) production within the penis were assessed. *In vitro* corpus cavernosum strips preparation was performed to evaluate the nitric oxide (NO) bioavailability. CB_2_ protein expression was shown in cavernosal endothelial and smooth muscle cells of wild type and hypercholesterolemic mice. Treatment with JWH-133 reduced ROS production and NADPH-oxidase expression in hypercholesterolemic mice penis. Furthermore, JWH-133 increased endothelial NO synthase expression in the corpus cavernosum and augmented NO bioavailability. The decrease in oxidative stress levels was accompanied with a reduction in corpus cavernosum collagen content. In summary, CB_2_ activation decreased histological features, which were associated with erectile dysfunction in hypercholesterolemic mice.

## 1. Introduction

Penile erection occurs when there is a relaxation of the smooth muscle cells in cavernosal arterioles and surrounding sinuses, resulting in increased blood flow into the penis associated with a pressure-dependent veno-occlusive mechanism within the sinuses controlling the blood outflow [1, 2]. The major mechanism responsible for ED is an increase in the tone and/or contractility of the smooth muscle within the corpus cavernosum and penile arteries [3], which is mostly due to diminished production and function of nitric oxide (NO) and other vasoprotective factors, which is compounded by the exaggerated production of reactive oxygen species (ROS) and vasoconstrictors [4, 5].

Hypercholesterolemia is one of the most important risk factors for the development of ED [5]. This phenotype produces various functional and structural alterations in the vasculature, frequently leading to the development of atherosclerosis [1, 4]. These vascular changes alter tissue perfusion and can impair the ability of arteries to respond to vasodilators factors [5–7]. Chronically, this impairment may result in cavernosal fibrosis, which may lead to permanent ED [8, 9]. One of the mechanisms mediated by hypercholesterolemia involves the alteration of oxidative stress and endothelial function in the penis [10–14]. In fact, it has been reported that these conditions increased protein expression of NAD(P)H oxidase subunits and induced eNOS uncoupling in the corpus cavernosum, resulting in increased oxidative stress and endothelial dysfunction [5].

The cannabinoid system is involved in a variety of pathophysiological processes including inflammation [15, 16], atherosclerosis [17, 18], obesity [19], cardiovascular disease [20–23], and erectile function [24, 25]. It has been shown that the endocannabinoid anandamide potentiates the neurogenic relaxation of rat corpus cavernosum, possibly through either CB_1_ or vanilloid receptors [25]. Others observed that both CB_1_ and CB_2_ receptors activation potentiated neurogenic relaxation of rabbit corpus cavernosum [26]. On the other hand, it was observed that anandamide inhibits neurogenic relaxation of corpus cavernosum of human and primates [27]. These data suggest a potential peripheral mechanism for cannabis-related modulations of sexual function; however, the role of the cannabinoid system on erectile function is still unclear.

Recently, we have shown that the CB_2_ selective agonist JWH-133 induces an atheroprotective effect in apolipoprotein-E-knockout (ApoE^−/−^) mice by improving intraplaque inflammation and vulnerability [28]. Therefore, we sought to investigate also the effects of this selective CB_2_ activation on oxidative stress and fibrosis within the corpus cavernosum in these hypercholesterolemic animals prone to develop atherosclerosis [28].

## 2. Materials and Methods

### 2.1. Experimental Design

In this investigation, the hypercholesterolemic ApoE^−/−^ mice model was used which is a well-established model for hypercholesterolemia inducing ED [7, 29–31]. This mouse model demonstrates erection deficiency, which has been related to an increased oxidative stress, reduction of penile endothelial function, and cavernosal fibrosis [29, 30]. This phenotype is closely related to human ED associated with hypercholesterolemia [1] and therefore is a suitable model for the present study. Male ApoE^−/−^ mice in a C57BL/6J background were obtained from Jackson Laboratories. Animals at 15–20 weeks of age were randomly assigned to receive either vehicle (Tocrisolve 100, Tocris Bioscience) or selective CB_2_ agonist JWH-133 (Tocris Bioscience, Bristol, UK) treatment. During an 11-week experimental period, all animals were fed a Western type diet consisting of 15% (wt/wt) cocoa butter and 0.25% (wt/wt) cholesterol (Diet W; abDiets). In the last 3 weeks of this experimental protocol, mice were intraperitoneally injected with JWH-133 (5 mg/kg/day for 5 consecutive days per week) or respective vehicle control. Age-matched wild type mice were used as additional controls. Twenty-four hours after the last drug administration, the animals were euthanized with the injection of ketamine 100 mg/Kg and xylazine 10 mg/Kg, and blood samples were collected by cardiac puncture for serum extraction immediately following cardiac puncture; the penis was removed and snap-frozen in liquid nitrogen and stored at –80°C for protein measurements or frozen in cryoembedding medium for histological analysis. This animal study was approved by the local ethics committee and Swiss regulatory authorities and conformed to the Helsinki Declaration.

### 2.2. Immunostaining in ApoE^−/−^ Mouse Penis

Six *μ*m cryosections from mice penes were fixed in acetone at room temperature and immunostained with the following antibodies: anti-CB_2_ (1 : 100, cat number sc-25494, Santa Cruz Biotechnology, Inc.) or anti-CB_2_ (1 : 100, cat number 301550, Cayman Chemical, Inc.), anti-*α*-actin (1 : 100, cat number sc-32251, Santa Cruz Biotechnology, Inc.), anti-PECAM (1 : 100, cat number sc-1506, Santa Cruz Biotechnology, Inc.), anti-rabbit IgG conjugated with Alexa Fluor-555 secondary antibody (1 : 400, cat number A31572, Invitrogen, Inc.), anti-mouse IgG conjugated with Alexa Fluor-647 secondary antibody (1 : 400, cat number A31571, Invitrogen, Inc.), and anti-goat IgG conjugated with Alexa Fluor-488 secondary antibody (1 : 400, cat number A21467, Invitrogen, Inc.). The negative control for CB_2_ receptor was performed using a specific blocking peptide (Cayman Chemical, cat number 301550). The slides were examined on a Confocal microscope equipped with a digital imaging system (Carl Zeiss LSM 700). 

### 2.3. Detection of Reactive Oxygen Species (ROS) in Corpus Cavernosum

To detect ROS (superoxide) production in the mice corpus cavernosum, cryosections were stained with dihydroethidium (DHE; Sigma-Aldrich, USA, cat number 37291). The cryosections (6 *µ*m) were allowed to thaw at room temperature and sequentially washed with PBS. Later, the sections were stained with DHE at 2 *µ*mol/L in PBS for 20 minutes at 37°C in the dark [32]. The slices were washed with PBS and examined on a confocal microscope. DHE fluorescence intensity of acquired digital images was quantified by ImageJ software (NIH).

### 2.4. Total Nitrite and Nitrate Assay in Serum

Nitrite (NO_2_^−^) and nitrate (NO_3_^−^) were measured as indirect measurements of nitric oxide (NO) content in mouse serum using a commercially available kit (Griess assay; R&D Systems, cat number KGE001). Prior to conducting the assay, the serum was filtered using a 10,000 molecular weight cut-off filters (Millipore, cat number UFC501096). The assay was performed as described in the instruction manual. Each sample was run as a technical triplicate.

### 2.5. Western Blotting of ApoE^−/−^ Mouse Penis

The protein expression of endothelial nitric oxide synthase (eNOS), nicotinamide adenine dinucleotide phosphate-oxidase (NADPH) subunit p47-phox (p47), and CB_2_ were quantified by western blotting. Forty micrograms of protein extracted from ApoE^−/−^ mice penises were run on a 10% SDS-PAGE gel, and the proteins were transferred onto a polyvinylidene fluoride membrane. After 1 hour of blocking with 1% casein in Tris-buffered saline-Tween, the membranes were probed with one of the following primary antibodies: anti-eNOS (1 : 200, cat number sc-654, Santa Cruz Biotechnology, Inc.); anti-p47 (1 : 200, cat number sc-7660, Santa Cruz Biotechnology, Inc.); and anti-CB_2_ receptor (1 : 200, cat number 25494, Santa Cruz Biotechnology, Inc.). Membranes were washed 3 times for 10 minutes in Tris-buffered saline-Tween and incubated with anti-rabbit IgG conjugated with Alexa Fluor-647 secondary antibody (1 : 3000, cat number A31573, Invitrogen, Inc.) for 2 hours at room temperature. After a series of final washes, the blots were detected using a fluorescence detector (Odyssey Imaging System, Li-Cor Biosciences).

### 2.6. Functional Studies in Cavernosal Tissue

After euthanasia, penes were excised and dissected in Krebs-Henseleit buffer (mmol/L: NaCl 110.8, KCl 5.9, NaHCO_3_ 25.0, MgSO_4_ 1.07, CaCl_2_ 2.49, NaH_2_PO_4_ 2.33, and glucose 11.51). The tunica albuginea was removed and one crural strip preparation was obtained from each corpus cavernosum (two crural strips from each penis). Cavernosal strips were mounted in isolated organ chamber system containing Krebs-Henseleit buffer at 37°C and continuously aerated with a mixture of 95% O_2_ and 5% CO_2_. The mechanical activity was recorded isometrically by a force transducer (ADInstruments, Colorado Springs, CO, USA). The tissue was stretched to a passive force of 3.0 mN and allowed to equilibrate for 60 min, and the solutions were replaced every 10 to 15 minutes. Changes in isomeric force were recorded using a PowerLab/8SP data acquisition system (Chart software, version 5.0; ADInstruments, Colorado Springs, CO, USA).

A dose-response relaxation was induced by acetylcholine (ACh, 10^−9^ mol/L to 10^−6^ mol/L) in strips preconstricted with phenylephrine (10^−5^ mol/L). Additionally, a dose-response curve for phenylephrine (at 10^−8^ mol/L to 10^−4^ mol/L) was performed in the presence or absence of the nitric oxide synthase (NOS) inhibitor, L-NAME (10^−4^ mol/L) to evaluate the NO basal production [33]. After the final concentration of phenylephrine, ACh at 10^−5^ mol/L was added to confirm the inhibition of NOS by L-NAME.

### 2.7. Sirius Red Staining for Collagen Content in Mouse Penis

Mouse penis sections (6 *µ*m) were rinsed with water; nuclei were stained with Weigert's hematoxylin for 10 minutes, washed in tap water, and incubated with 0.1% sirius red (Sigma Chemical Co., St. Louis, MO) in saturated picric acid for 60 minutes. Sections were rinsed twice with 5% acetic acid in water for 10 seconds and then immersed in absolute ethanol three times before clearing in xylene twice and cover-slipping. The sections were photographed with identical exposure settings under light microscopy. Quantifications of collagen content were performed with ImageJ software. Data was calculated as smooth muscle cells and collagen content ratio.

### 2.8. Data Analysis

The results are expressed as mean ± SEM. Statistical analyses for western blot, Sirius red, and DHE staining were performed using the one-way ANOVA followed by Bonferroni post-test. Statistical analyses for Griess assay were done using Student-*t*-test. Finally, the statistical analyses of the corpus cavernosal tissue bath experiments were performed using two-way ANOVA followed by Bonferroni post-test. A value of *P* < 0.05 was considered significant.

## 3. Results

### 3.1. CB_2_ Receptor Is Expressed in Mouse Penis

**Figure 1:**
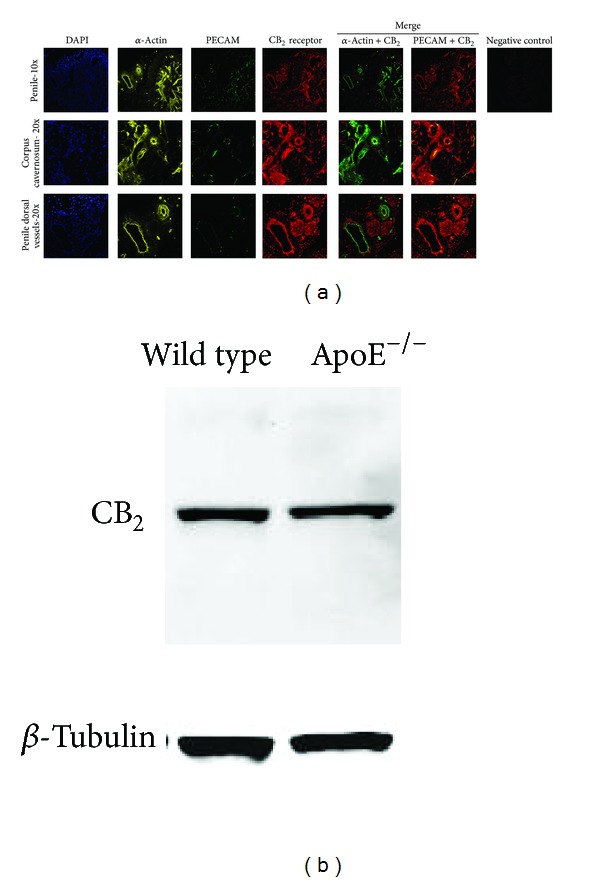
CB_2_ receptor is expressed in mouse penis. Immunostaining and western blotting analysis was used to detect CB_2_ receptor on penis from wild type and ApoE^−/−^ mice. (a) CB_2_ was strongly colocalized with *α*-actin (smooth muscle cells marker) as well as PECAM (endothelial cell marker) in the corpus cavernosum and dorsal vessels of wild type and ApoE^−/−^ mice. The graph shows representative images obtained from 8 different animals. (b) CB_2_ in the mouse penis was confirmed by western blotting which revealed a specific single band at the expected molecular weight (approximately 45 kDa). The graph shows a representative gel of western blotting showing the expression of CB_2_ in penis from wild type and ApoE^−/−^ mice from 4 independent experiments.

Two different commercial antibodies were used to detect CB_2_ receptor in the corpus cavernosum of wild type and ApoE^−/−^ mice. Both antibodies showed consistent results, and CB_2_ receptor was colocalized with smooth muscle cells (*α*-actin positive cells) and endothelial cells (PECAM positive cells) of corpus cavernosum and dorsal vessels of wild type and ApoE^−/−^ mice (Figure 1(a)). Additionally, the expression of CB_2_ in the mouse penis was confirmed by western blotting which revealed a specific single band at the expected molecular weight (approximately 45 kDa, Figure 1(b)). No difference in CB_2_ expression was observed between wild type mice and untreated and JWH-133-treated ApoE^−/−^ mice.

### 3.2. JWH-133 Reduces ROS Content in ApoE^−/−^ Mouse Penis

**Figure 2:**
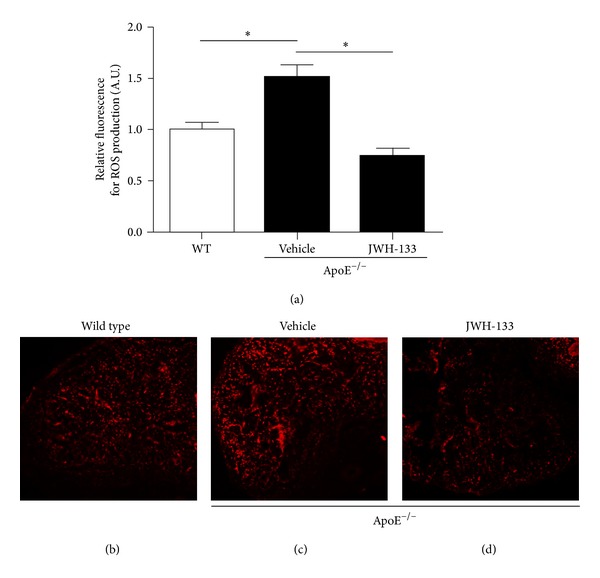
Selective CB_2_ activation decreases ROS content in corpus cavernosum. JWH-133 decreases ROS content in corpus cavernosum of ApoE^−/−^ mice. (a) Quantification of ROS content. ((b)–(d)) Representative photomicrographs of penis sections showing the ROS production using DHE staining in wild type mice (b) and untreated (c) and treated (d) ApoE^−/−^ mice. *P < 0.05 (one-way ANOVA followed by Bonferroni posttest). Each column represents the mean ± SEM (*n* = 6) of relative fluorescence in arbitrary unity (A.U.).

**Figure 3:**
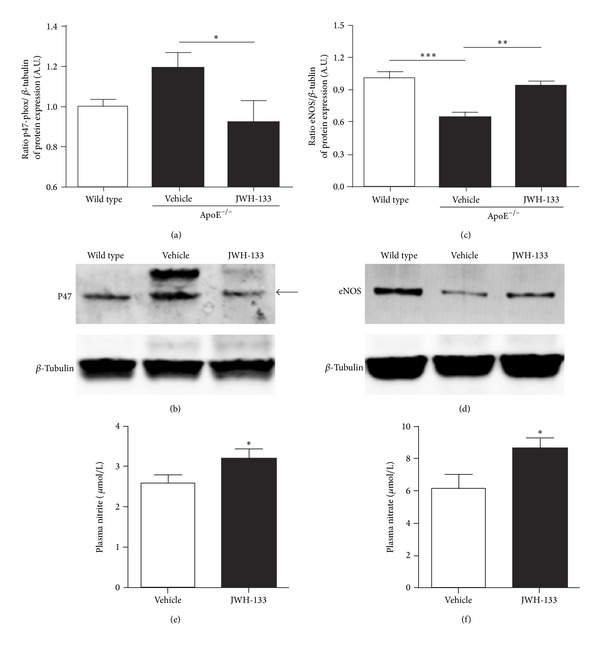
CB_2_ activation reduces NADPH oxidase and increases eNOS protein expression. ((a)-(b)) JWH-133 treatment decreased p47 levels into the penis of ApoE^−/−^ mice. (a) Quantification of the Western blotting data. (b) Representative gel. Data were normalized using *β*-tubulin. ((c)-(d)) Three weeks of JWH-133 treatment increased eNOS protein levels into ApoE^−/−^ mice penis. (c) Quantification of the western blotting data. (d) Representative gel. Data were normalized using *β*-tubulin. *P < 0.05 (One-way ANOVA followed by Bonferroni post-test). Each column represents the mean ± SEM of relative protein expression in arbitrary unity (A.U.) from 3 independent experiments. ((e)-(f)) The measurement of the NO stable metabolites, nitrite and nitrate, was determined by Griess assay. JWH-133 treatment significantly increased nitrite and nitrate levels, in serum from ApoE^−/−^ mice. Each column represents the mean ± SEM (*n* = 8) of serum nitrite and nitrate levels (*μ*mol/L).

Evidence indicates that ROS plays an important role in the development of erectile dysfunction in hypercholesterolemia [10]. Thus, we evaluated the effect of JWH-133 treatment as compared to vehicle treatment on ROS production in penis from ApoE^−/−^ hypercholesterolemic mice [30]. Lipid profile at sacrifice was comparable in both mouse treatment groups (Table 1). As shown in Figure 2, ROS production was augmented in ApoE^−/−^ mice penis. Interestingly, JWH-133 treatment significantly reduced ROS production in the corpus cavernosum of ApoE^−/−^ mice to a similar level of wild type mice. Moreover, this effect was associated with a reduction in the protein expression of the NADPH oxidase subunit p47-phox (Figures 3(a) and 3(b)).

### 3.3. JWH-133 Increases eNOS Expression and NO Basal Production in Mouse Penis

The NO is the major effector on penile erection, and its reduced availability is positively correlated with ED in hypercholesterolemia [5, 34]. As expected, eNOS protein expression was reduced in ApoE^−/−^ mice compared to wild type mice. Interestingly, JWH-133 treatment significantly attenuated its reduction in ApoE^−/−^ mice penis (Figures 3(c) and 3(d)). Additionally, JWH-133 increased the levels of the stable NO metabolites, NO_2_^−^ and NO_3_^−^, in ApoE^−/−^ mouse serum (Figures 3(e) and 3(f)).

**Figure 4:**
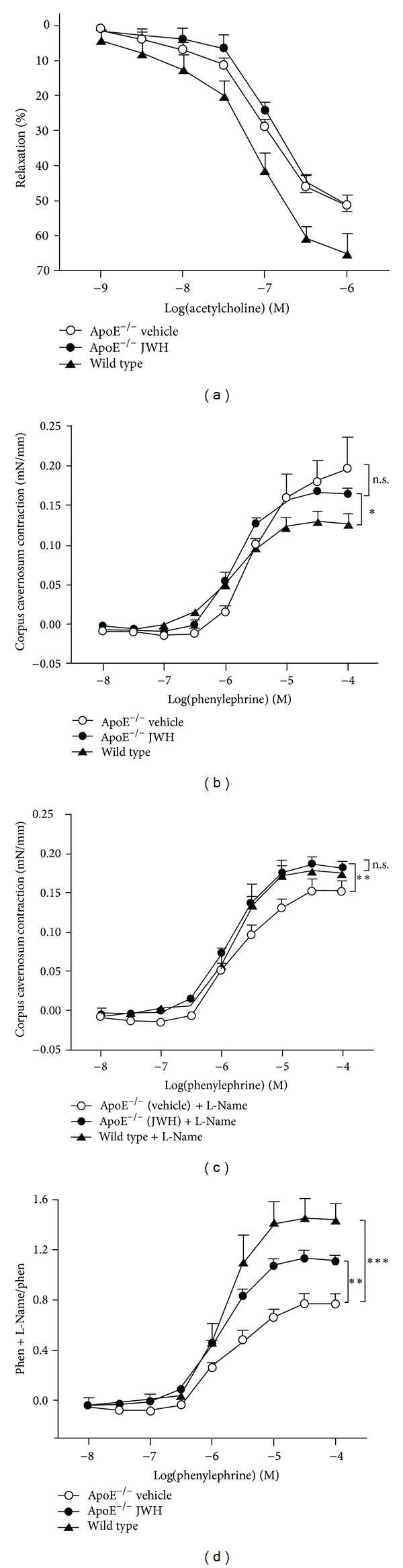
The treatment with JWH-133 increased NO bioavailability in the corpus cavernosum of ApoE^−/−^ mice. Relaxation produced by increasing cumulative concentrations of acetylcholine (a) in cavernosal strip from wild type mice and untreated and JWH-133-treated ApoE^−/−^ mice. ((b)-(c)) Constriction induced by cumulative concentrations of phenylephrine in the presence (c) or absence (b) of NOS inhibitor (L-NAME) in cavernosal strip. (d) Normalized curve response of phenylephrine in the presence of L-NAME by phenylephrine response at 10^−6^ mol/L. *P < 0.05, **P < 0.01, and ***P < 0.001 (two-way ANOVA followed by the Bonferroni multiple comparison test). Each point represents the mean ± SEM (*n* = 7 to 10). n.s.: nonsignificant.

In the *in vitro* corpus cavernosum strip preparation, we observed that the contraction induced by phenylephrine was lower in wild type mice compared to untreated ApoE^−/−^ mice (Figure 4(b)). Furthermore, when the cavernosal strips were preincubated with L-NAME, the contractions produced by phenylephrine were higher in wild type mice compared to untreated ApoE^−/−^ mice (Figure 4(c)), indicating that NO basal production is reduced in ApoE^−/−^ mice compared to wild type mice. Moreover, we observed that phenylephrine contractile response was not different between JWH-133-treated and untreated ApoE^−/−^ mice cavernosal strips (Figure 4(b)). Interestingly, under preincubation with L-NAME, the contraction induced by phenylephrine was more robust in JWH-133-treated than untreated mice (Figure 4(c)), showing that punctual blockage of NOS influences more phenylephrine response in JWH-133-treated ApoE^−/−^ mice than untreated mice, indicating an increase in NO basal production in the penis by JWH-133 treatment. The normalization of the phenylephrine plus L-NAME response with the phenylephrine response evidenced even more this effect (Figure 4(d)). Intriguingly, no differences were observed in the relaxant response produced by ACh in JWH-133-treated and untreated ApoE^−/−^ mice (Figure 4(a)). These data suggest that JWH-133 treatment may increase the NO basal production in the erectile tissue but not the stimulated production. 

### 3.4. JWH-133 Treatment Decreases Penis Fibrosis in ApoE^−/−^ Mouse

**Figure 5:**
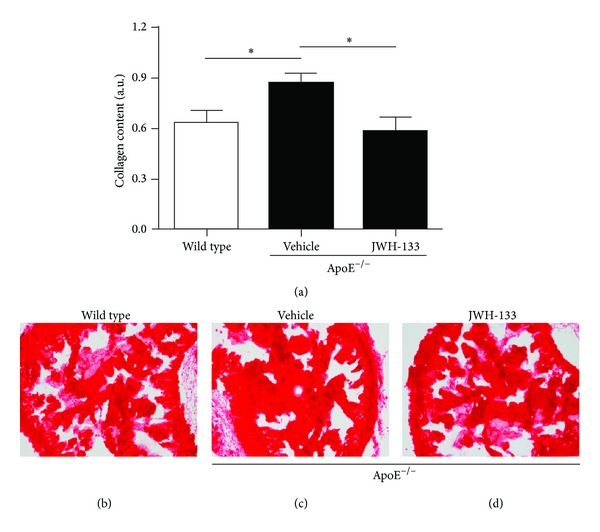
JWH-133 protects ApoE^−/−^ mouse corpus cavernosum against fibrosis. Collagen content was evaluated by sirius red staining in penis from wild type and ApoE^−/−^ mice treated or not with JWH-133. (a) Quantification of collagen content into the corpus cavernosum. ((b)–(d)) Representative photomicrographs of penis sections showing the corpus cavernosum of wild type mice (b) and untreated (c) and treated (d) ApoE^−/−^ mice. *P < 0.05 (one-way ANOVA followed by the Bonferroni post-test). Each column represents the mean ± SEM (*n* = 9) of smooth muscle cell/collagen content ratio in arbitrary unity (A.U.).

We also evaluated the effect of JWH-133 treatment on collagen deposition, since one of the consequences of oxidative stress is fibrosis [35]. Treatment with JWH-133 significantly reduced the collagen content in the corpus cavernosum (Figure 5).

## 4. Discussion

The major finding of this study is that the treatment with the selective CB_2_ agonist JWH-133 was associated with the reduction of penis oxidative stress and fibrosis in hypercholesterolemic mice. In particular, JWH-133 induced protective effects by decreasing ROS release and NADPH oxidase expression and by increasing NO basal production and eNOS expression in the ApoE^−/−^ mice penis. Furthermore, these effects were associated with a reduction of collagen deposition into corpus cavernosum.

The beneficial effects of CB_2_ improving the inflammatory profile have been already studied [15, 16]. This receptor is highly expressed in most types of immune cells and regulates immunity through different mechanisms [36]. However, the physiological role of this receptor remains controversial [17]. Recent studies have shown that CB_2_ receptor is also expressed by other cell types, such as nonparenchymal liver cells [37], cardiomyocytes [38], vascular smooth muscle cells [39], and endothelial cells [40]. There is an evidence that CB_2_ activation has beneficial effects in animal models of chronic degenerative diseases, such as atherosclerosis [17, 28] and liver fibrosis [37, 41, 42], by reducing inflammatory, oxidative, and fibrotic processes. Here, we provided evidence that CB_2_ also protects penile function and structure against degenerative consequences of hypercholesterolemia. The functional actions of CB_2_ receptor in the erectile tissues were first demonstrated by Vural and colleagues [26] in rabbit corpus cavernosum. They found that JWH-015, another CB_2_ agonist, potentiated electrical field stimulation inducing cavernosal strip relaxation while AM-630, a selective inverse CB_2_ agonist attenuated its effect [26]. Others observed that anandamide, a nonselective endogenous CB_1_ and CB_2_ agonist, potentiated the neurogenic relaxation of rat corpus cavernosum, which was blocked by a selective CB_1_ receptor antagonist and a vanilloid receptor antagonist but not by a CB_2_ receptor antagonist [25]. Moreover, in that study, CB_2_ receptor protein expression was not detected in the rat corpus cavernosum [25]. On the other hand, Gratzke and colleagues [27] have shown that anandamide depressed the neurogenic relaxation of human and primate corpus cavernosum strips, which was consistent with epidemiologic data associating the use of cannabis with sexual dysfunction in men [43]. Moreover, CB_1_ and CB_2_ receptors were detected in nerve fiber of human and primate corpus cavernosum [27]. Thus, these data suggest that cannabinoid receptors expression and function in the penis may vary between species. Another possibility is the formation of CB_1_-CB_2_ receptor heteromer recently reported [44]. The heteromer activity stimulated by CB_1_ agonists or CB_2_ agonists may be blocked by either CB_1_ antagonists or CB_2_ antagonists, showing a bidirectional cross-antagonism phenomenon [44]. In the present study, CB_2_ protein expression in mouse penis was shown by western blotting, which revealed a specific single band at the expected molecular weight (approximately 45 kDa) for CB_2_. Moreover, CB_2_ was co-localized with endothelial and smooth muscle cells of corpus cavernosum and penile dorsal vessels, which is in keeping with previous studies showing the expression of this receptor is these cell types [39, 40]. Similarly to the studies mentioned above, in the present study, the detection of CB_2_ was assessed by immunoassays. Despite of the clear specific single band observed by western blotting, due to the limitations of immunoreactions, the immunostaining for CB_2_ was performed using two different commercial first antibodies while a blocking peptide was used as negative control. Both first antibodies revealed consistent results. These data indicate that CB_2_ is expressed in mouse penis, suggesting a peripheral mechanism for CB_2_-related modulation of sexual function.

In the present study, the beneficial action of CB_2_ against hypercholesterolemia inducing erectile tissue damages was assessed using the selective agonist JWH-133. This compound is a potent CB_2_ receptor agonist, with a Ki of 3.4 nM and 200-fold selectivity for the CB_2_ receptor over CB_1_ receptors [45]. The dose used in our study was selected on the basis of previous studies on the same mouse background [25, 30, 46], showing a selective CB_2_ receptor activation. Thus, the activation of CB_1_ by JWH-133 in our study is not probable; however, this possibility may not be completely ignored. 

Evidence reveals that the increase of oxidative stress mediated through ROS may be central to impaired cavernosal function in ED [5, 32, 47]. In fact, the reduced erectile response of hypercholesterolemic ApoE^−/−^ mice has been associated with an increase in corpus cavernosum ROS content [48], which was confirmed by our results. Musicki and coworkers showed that the impaired erectile response of LDLR-null hypercholesterolemic mice was associated with increased protein expressions of NADPH oxidase subunits p67phox, p47phox, and gp91phox [5]. In our study, we observed that pharmacological activation of CB_2_ by JWH-133 reduced ROS content and the expression of p47phox in the corpus cavernosum of ApoE^−/−^ mice. Therefore, our results suggest that CB_2_ activation reduces oxidative stress which is in accordance with the previous studies [17, 23]. Conversely, CB_1_ receptor activation is related to the augmentation of oxidative stress as well as other depressive effects [49–52]. In fact, selective CB_1_ antagonists have been speculated as a potential tool for the treatment of cardiovascular disease [32, 49]. In the present study, the role of CB_1_ was not addressed. Few studies have shown contradictory effects of CB_1_ activation in erectile tissues [24, 25, 27]. Thus, future studies addressing the role of CB_1_ in the penis will give important data to understand the role of the cannabinoid system in the penile function. 

Many cannabinoid agonists may produce protective effects and reduce ROS through receptor-independent antioxidant mechanisms. A priori, this capability seems to be inherent to compounds such as the plant-derived cannabinoids whose chemical structure with phenolic groups enables them to act as ROS scavengers [53]. JWH-133 is a synthetic CB_2_ agonist that does not possesses phenolic group; thus, its action as ROS scavenger is not probable. However, there is no study addressing the antioxidant effect of this compound. This possible mechanism is an interesting issue that needs to be addressed in future studies. 

It is well known that NO plays a crucial role in erectile response, and impaired NO bioactivity is a major pathogenic mechanism of erectile dysfunction [4, 54]. The NO/cyclic guanosine monophosphate pathway is considered the most important intracellular mechanism responsible for smooth muscle relaxation leading to erection [55]. In our study, we found that JWH-133 treatment increased the stable NO metabolites nitrite/nitrate serum levels. Since these results represent a systemic vascular increase in NO production and do not clarify the effect of JWH-133 modulating NO in erectile tissues, we evaluated the local protein levels of eNOS. The eNOS protein expression in the penis was increased by JWH-133 treatment, suggesting that this compound also regulates NO bioavailability in the penis. Additionally, to expand this point, we observed that the contraction induced by phenylephrine was more robust in the presence of L-NAME in JWH-133-treated compared to untreated mice while the relaxation produced by ACh in preconstricted cavernosal strip was not changed comparing treated and untreated ApoE^−/−^ mice. These *in vitro* data suggest that JWH-133 increased the basal production of NO. but not the stimulated production. 

Chronically, the increased oxidative stress in the erectile tissue contributes to the augmented collagen deposition and penile fibrosis, which worsen erectile function and may lead to a permanent condition of ED [9, 35]. In fact, it was shown that the ApoE^−/−^ mouse has an increased penile fibrosis, which was associated with oxidative stress [56]. In our study, we observed that JWH-133 also reduced collagen content in the corpus cavernosum. The mechanism of this effect appears to be, at least in part, due to a reduction of ROS production.

In the present study, the mechanism implied in the protective action of JWH-133 against hypercholesterolemia inducing penile oxidative stress and fibrosis was not deeply investigated. One may argue that the beneficial effect of JWH-133 treatment could be due to the reduction of local cell infiltration. In fact, we have previously shown that JWH-133 treatment reduced cell infiltration in atherosclerotic plaques of ApoE^−/−^ [30]. Therefore, we investigated the presence of macrophage and neutrophil in the corpus cavernosum of wild type, JWH-133-treated and untreated ApoE^−/−^ mice. Cell infiltration was evaluated by immunostaining using specific markers (CD68 and neutrophil elastase). While control slides of mice spleen showed a positive staining, none or only few cells were detected in penis samples. Moreover, no differences were observed between the groups (data not shown). In our animal model, the ApoE^−/−^ mice received western type diet for 11 weeks. This period was sufficient to develop atherosclerotic plaque [30]; however, it appears to be a short period to detect cell infiltration in the corpus cavernosum. Thus, it appears that in our study the beneficial effects of JWH-133 on erectile tissues were not related to cell infiltration reduction.

Of note, as we have documented previously [30], JWH-133 treatment did not change glucose, triglycerides, total cholesterol, and LDL and HDL serum levels in ApoE^−/−^ mice (supplemental data), indicating that the effects promoted by JWH-133 observed in this study were not related to lipid profile changes.

## 5. Conclusion

In summary, these findings demonstrate that the selective CB_2_ agonist, JWH-133, decreases ROS production in the corpus cavernosum of hypercholesterolemic mice. Moreover, it suggests that JWH-133 treatment increases basal NO production within the penis. Furthermore, these effects were associated with a reduction in the corpus cavernosum collagen content. These data suggest that CB_2_ activation improves the oxidative stress and fibrosis deposition in hypercholesterolemic condition.

## Figures and Tables

**Table 1:** Mouse serum lipid profile at sacrifice.

Serum lipid profile (mmol/L)	Vehicle-treated mice	JWH-133-treated mice	*P* value
Total cholesterol	24.03 ± 2.432	22.30 ± 2.102	0.5988
LDL	18.11 ± 1.248	17.21 ± 1.421	0.6386
HDL	3.464 ± 0.276	3.585 ± 0.209	0.7482
Triglycerides	1.066 ± 0.124	0.986 ± 0.209	0.7382
Fatty-free acid	0.475 ± 0.079	0.503 ± 0.046	0.7670

Data are expressed as mean ± SEM.

*P* value calculated according to unpaired *t*-test.
